# Vitamin D deficiency in critically ill patients with traumatic injuries

**DOI:** 10.1186/s41038-016-0054-8

**Published:** 2016-10-17

**Authors:** Roland N. Dickerson, Jonathan R. Van Cleve, Joseph M. Swanson, George O. Maish, Gayle Minard, Martin A. Croce, Rex O. Brown

**Affiliations:** 1 Department of Clinical Pharmacy, University of Tennessee Health Science Center, 881 Madison Avenue, Suite 345, Memphis, 38163 TN USA; 2 Department of Surgery, University of Tennessee Health Science Center, 910 Madison Avenue, 2nd Floor, Memphis, 38163 TN USA

**Keywords:** Vitamin D, Deficiency, Depletion, Trauma, Injury, Parenteral nutrition, Enteral nutrition, African-American, Obesity

## Abstract

**Background:**

Vitamin D depletion has been associated with increased rate of infections, lengthened hospital stay, and worsened mortality for critically ill patients. The purpose of this study was to evaluate the prevalence and variables associated with vitamin D deficiency in critically ill patients with severe traumatic injuries.

**Methods:**

Critically ill adult patients admitted to the trauma intensive care unit (ICU) between June 2013 and June 2014, referred to the nutrition support service for enteral or parenteral nutrition, and had a serum 25-hydroxyvitamin D (25-OH vitamin D) concentration determination were retrospectively evaluated. Patients were stratified as vitamin D sufficient, insufficient, deficient, or severely deficient based on a 25-OH vitamin D concentration of 30–80, 20–29.9, 13.1–19.9, and ≤13 ng/mL, respectively.

**Results:**

One hundred and twenty-one patients out of 158 (76 %) patients were vitamin D deficient or severely deficient. Thirty-one patients (20 %) were insufficient and 6 (4 %) had a normal 25-OH vitamin D concentration. 25-OH vitamin D was determined 7.5 ± 5.1 days after ICU admission. African-Americans had a greater proportion of patients with deficiency or severe deficiency compared to other races (91 versus 64 %, *P* = 0.02). Penetrating gunshot or knife stab injury, African-American race, and obesity (elevated body mass index) were significantly associated with vitamin D deficiency or severe deficiency: OR 9.23 (1.13, 75.40), 4.0 (1.4, 11.58), and 1.12 (1.03, 1.23), *P* < 0.05, respectively.

**Conclusions:**

The majority of critically ill patients with traumatic injuries exhibit vitamin D deficiency or severe deficiency. Penetrating injuries, African-American race, and obesity are significant risk factors for deficiency. Severity of injury, extent of inflammation (elevated C-reactive protein concentration), or hospital admission during the winter season did not significantly influence the prevalence of vitamin D deficiency.

## Background

Vitamin D is well known for its importance in calcium and phosphorus homeostasis and bone formation. Recent studies demonstrate that vitamin D may have an important role in modulating the innate and adaptive immune response to infectious pathogens including gram negative and positive bacteria, fungi, and mycobacteria [1]. Vitamin D deficiency in critically ill patients has been associated with increased risk of infectious complications and mortality [2–8] as it is an integral component to the production of cathelicidin and other antimicrobial proteins produced by macrophages and neutrophils [9]. The synthesis of these antimicrobial proteins is highly expressed at integumentary barrier sites such as the respiratory epithelium [9].

Unfortunately, data regarding vitamin D depletion in critically ill patients with severe multiple traumatic injuries are limited due to studies that have incorporated these patients as part of a larger population [10, 11], evaluated patients months after the acute phase of their injury [12, 13], or evaluated non-critically ill patients post injury [14, 15]. Since infections, especially epithelial barrier site infections such as pneumonia, are common complications leading to death in approximately 20 % of critically ill trauma patients [16], it would be important to ascertain if vitamin D deficiency is prevalent in this population. The intent of this study was to ascertain the incidence and risk factors for the development of vitamin D deficiency among critically ill trauma patients who required enteral or parenteral nutrition therapy and who were anticipated to have a prolonged intensive care unit (ICU) stay.

## Methods

### Design and patient selection

Adult patients, at least 18 years of age, with severe traumatic injuries and admitted to the ICU of the Presley Trauma Center at Regional One Health in Memphis, Tennessee, and referred to the Nutrition Support Service (NSS) for enteral nutrition (EN) or parenteral nutrition (PN) were eligible for study inclusion. The center is designated as a level 1 trauma center and serves the mid-south region of the USA (western Tennessee, northern Mississippi, Arkansas). Patients had a serum 25-hydroxy vitamin D (25-OH vitamin D) determination while in the ICU following patient referral to the NSS as part of their routine metabolic assessment. Patients excluded from study entry were those known to receive vitamin D beyond a daily multivitamin supplement or those who received chronic glucocorticoids, HIV medications [17], or other medications known to increase vitamin D catabolism [18, 19] prior to hospital admission. Females who were pregnant were also excluded.

Study candidates were identified from the Nutrition Support Service monitoring records from June 2013 to June 2014. Winter was defined as November through March based on the presence of reduced skin exposure to ultraviolet light [11, 20]. Patients’ electronic medical record and nutrition support service record were retrospectively reviewed for data retrieval. Injury severity score [21] (ISS) was scored by trained nurses according to the American Association for the Surgery of Trauma scales for anatomic injury severity and recorded within the data repository of the Tennessee trauma registry at Regional One Health. The study was approved and conducted in accordance with the guidelines established by the University of Tennessee Health Science Center Institutional Review Board and the Regional One Health Office of Medical Research (study reference number 14-02924-XM). Since this was a retrospective, non-interventional study, requirement for written informed consent was waived.

### Nutritional regimen and vitamin D intake

Patients were preferentially given enteral nutrition by a nasogastric or orogastric feeding tube whenever possible. Patients were assigned energy and protein goals of 25–32 kcal/kg/d and 2–2.5 g/kg/d per day, respectively [22]. If the patient’s ISS was greater than 20 and enteral access was viable, an enteral formula (1.3 kcal/mL, 78 g protein/L) containing glutamine, arginine, dietary nucleotides, and ω-3 fatty acids was initiated [23]. Other enteral-fed patients received either a polymeric, high-protein formula (1 kcal/mL, 62 g protein/L or 1 kcal/mL, 93 g protein/L) or a diabetic formula (1.2 kcal/mL, 60 g protein/L). Commercial enteral formulas contained 400–533 IU of vitamin D_3_ per 1000 kcals. Liquid protein supplements were also given per discretion of the NSS [22]. Patients requiring parenteral nutrition received an individualized prescription to meet target energy and protein goals as a total nutrient admixture via central venous access. Patients who required PN received 200 IU of vitamin D_3_ daily as part of the intravenous multivitamin additive to the PN admixture. Daily EN and PN intakes were recorded for the first fourteen days of nutrition therapy. Pre-resuscitation body weight was used to determine target nutritional goals. Blood glucose concentrations were maintained between 70 and 150 mg/dL with the use of intravenous regular human insulin therapy as previously described [24–26].

### Measured variables

Serum 25-OH vitamin D concentrations were obtained at 0100 via a venous catheter approximately weekly while the patient remained in the ICU and required PN or EN. Serum 25-OH vitamin D concentrations were assayed by the hospital laboratory via an automated chemiluminescent microparticle immunoassay (Architect System, Abbott Laboratories, Abbott Park, IL, USA). The detection limit was 8 ng/mL (20 nmol/L). Intra-assay and inter-assay coefficient of variations were between 1.4 and 4.6 % for concentrations ranging from 18 to 78 ng/mL (45 to 195 nmol/L) [27]. Verification studies for accurate concentration determination ranged from 13 to 96 ng/mL (32 to 240 nmol/L) per the manufacturer recommendations [28]. Other laboratory tests were ordered by the patient’s primary service or the NSS and performed by the hospital laboratory as part of the patient’s routine clinical care.

Classification of vitamin D insufficiency and deficiency was based on the recommendations of the Institute of Medicine [18]. A normal 25-OH vitamin D concentration was established as 30 to 80 ng/mL. Patients with a 25-OH vitamin D of 20–29.9 ng/mL were considered vitamin D insufficient whereas those with a 25-OH vitamin D of 13.1–19.9 ng/mL were categorized as deficient and ≤13 ng/mL as severely deficient [11, 18]. Patients with a serum 25-OH vitamin D concentration determination <20 ng/mL were prescribed liquid ergocalciferol 50,000 IU once to three times weekly via the feeding tube as previously described [29]. Patients who received parenteral nutrition did not receive additional liquid enteral vitamin D supplementation beyond intravenous maintenance requirements in the parenteral nutrition solution until enteral access was established [29]. Only serum 25-OH vitamin D measurements that were obtained prior to enteral liquid vitamin D supplementation were used in this analysis.

### Statistical analysis

Data analysis was conducted using SigmaPlot for Windows, version 11.2 (Systat Software, Point Richmond, VA) and SPSS version 22 (IBM Corporation, Chicago, IL). The significance testing and reported probability values (*P*) were two-sided for all variables. A *P* value of ≤0.05 was defined as statistically significant. Continuous data were expressed as mean ± standard deviation. The normality of the distribution of the data was evaluated by the Shapiro-Wilk test. Differences between vitamin D depletion groups for continuous variables were analyzed using one-way analysis of variance with post hoc pair-wise multiple comparisons by the Student-Newman-Keuls test for normally distributed data or Kruskal-Wallis one-way analysis of variance with pair-wise multiple comparison by Dunn’s method for non-normally distributed data. Categorical data were evaluated by chi-square analysis. The paired *t* test or Wilcoxon signed rank test was used for comparing two simultaneous 25-OH vitamin D concentrations within the same set of patients. A preliminary bivariate correlation analysis of all collected categorical and quantitative variables for the presence of vitamin D deficiency was conducted to determine which covariates to use in the binary logistic regression analysis. Those variables that achieved a statistically significant trend of *P* ≤ 0.15 were used in the multivariate model. Odds ratio (OR) was expressed as mean with 95 % confidence intervals.

## Results

### Patient characteristics

A total of 158 patients admitted to the trauma ICU were evaluated. The majority of patients were male (82 %), admitted to the hospital due to a motor vehicle collision (62 %), and survived (84 %). Patients exhibited an ISS consistent with severe injury, had a markedly elevated C-reactive protein (CRP) concentration, depressed serum prealbumin concentration, and most were adequate in body weight (body mass index ≥18.5 kg/m^2^). Patients exhibited a mean elevated CRP of 22 mg/dL with a concentration <10 mg/dL in only 32 patients (20 % of the total population). None of the patients had a normal CRP concentration (<1 mg/dL). The majority of patients were either Caucasian (47 %) or African-American (41 %) with the remaining patients of either Hispanic or Asian descent. Other patient characteristics among the vitamin D groups are given in Table 1.

**Table 1:** Patient characteristics

Variable	Normal	Insufficient	Deficient	Severely deficient	*P* ≤
25-OH vitamin D concentration range, ng/mL	30–80	20–29.9	13.1–19.9	≤13	
*N*	6	31	75	46	
Male/female, *n*/*n*	5/1	27/4	61/14	37/9	0.885
Race					
Caucasian, *n* (%)	5 (83 %)	22 (71 %)	34 (45 %)	13 (28 %)	0.004
African-American, *n* (%)	1 (17 %)	5 (16 %)	32 (43 %)	27 (59 %)	
Hispanic/other, *n* (%)	0 (0 %)	4 (13 %)	9 (12 %)	6 (13 %)	
Etiology for admission					
MVA, *n* (%)	1 (17 %)	21 (68 %)	48 (64 %)	18 (39 %)	0.001
GSW/KSW, *n* (%)	0 (0 %)	1 (3 %)	14 (19 %)	19 (41 %)	
Fall/Assault, *n* (%)	5 (83 %)	6 (19 %)	9 (12 %)	6 (13 %)	
Other, *n* (%)	0 (0 %)	3 (10 %)	4 (5 %)	3 (7 %)	
Age, years	60 ± 30	46 ± 22	45 ± 18	43 ± 17	0.243
Weight, kg	70 ± 20	85 ± 17	88 ± 23	87 ± 19	0.206
BMI, kg/m^2^	22 ± 5	27 ± 4	28 ± 7	29 ± 5	0.033
Injury severity score	25 ± 6	30 ± 14	27 ± 14	29 ± 13	0.399
C-reactive protein, mg/dL	20.4 ± 12.0	18.5 ± 12.0	22.1 ± 12.5	23.7 ± 11.9	0.380
Prealbumin, mg/dL	8.9 ± 5.6	9.9 ± 8.2	8.7 ± 4.1	8.5 ± 3.7	0.989
Alcohol abuse, *n* (%)	1 (17 %)	10 (32 %)	17 (23 %)	12 (26 %)	0.720
Admitted Nov–Mar, *n* (%)	3 (50 %)	10 (32 %)	29 (39 %)	24 (52 %)	0.300
Serum ionized calcium, mmol/L	1.19 ± 0.06	1.17 ± 0.06	1.18 ± 0.09	1.16 ± 0.07	0.407
Serum phosphorus, mg/dL	2.9 ± 0.3	3.8 ± 0.8	3.6 ± 1.0	3.6 ± 1.0	0.055
25-OH vitamin D, ng/mL	39.2 ± 7.3	23.2 ± 3.0	16.0 ± 2.2	12.8 ± 0.5	0.001
Hospital day of vitamin D determination, days	6.2 ± 2.9	9.8 ± 5.6	7.4 ± 5.3	7.1 ± 4.5	0.077

*BMI* body mass index, *GSW* gunshot wound, *KSW* knife stab wound, *Mar* March, *MVA* motor vehicle accident, *N* number of patients, *Nov* November, *OH* hydroxy

*Significantly different (*P* ≤ 0.05) from the other groups

### Prevalence and associated risk factors for vitamin D deficiency

The vast majority of patients (121, 77 %) were either vitamin D deficient or severely deficient. Forty-six patients (38 % of the total population) exhibited severe deficiency. Only six patients (4 %) had a normal serum 25-OH vitamin D concentration. The remaining 31 patients (19 % of the total population) were vitamin D insufficient. Age, sex, proportion of patients with a history of alcohol or illicit drug abuse, serum prealbumin concentration, or hospital day of the 25-OH vitamin D determination were not statistically different between vitamin D groups (*P* = NS, Table 1). Although there were no differences in body weight among the vitamin D groups, body mass index (BMI) was significantly lower for those with normal vitamin D concentrations compared to the other groups (*P* = 0.033; Table 1).

There was no significant difference in serum 25-OH vitamin D for those who were admitted in the winter season (*n* = 66) versus the other months (*n* = 92): 17.4 ± 7.0 versus 17.4 ± 5.5 ng/mL, respectively (*P* = 0.642). Eighty percent of patients admitted in the winter (53 out of 66) were vitamin D deficient compared to 74 % of patients (68 out of 92) admitted in the remainder of the year (*P* = 0.456). Thirty-six percent of patients (24 out of 66) admitted in the winter were severely vitamin D deficient compared to 24 % of patients (22 out of 92) admitted in the other months of the year (*P* = 0.128).

**Fig. 1:**
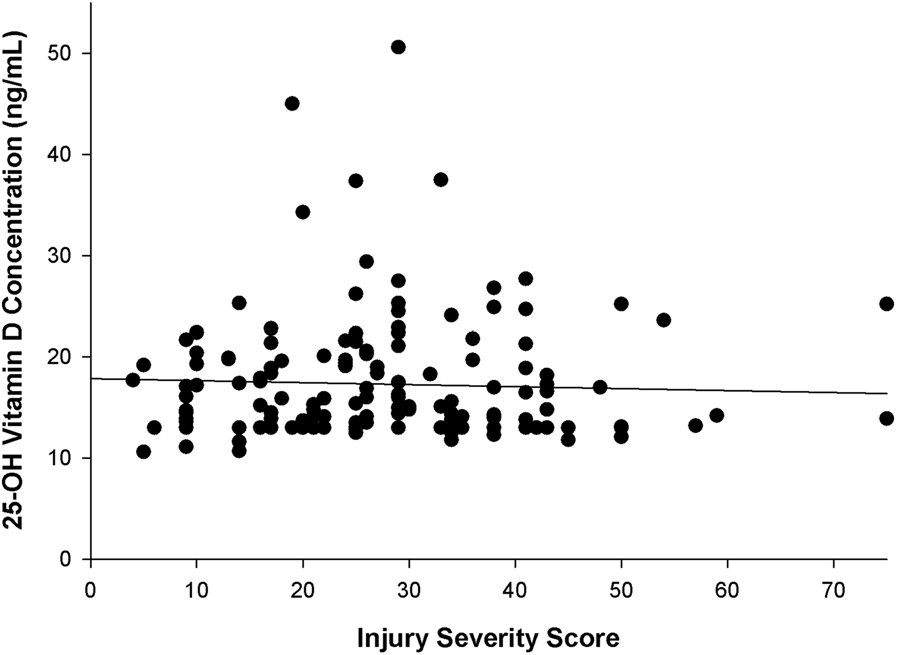
Relationship between serum 25-OH vitamin D concentration and severity of injury (*r* = 0.046, *P* = 0.571). The insignificant inverse linear relationship was expressed as 25-OH vitamin D concentration = 18.0 − (0.02 × injury severity score)

**Fig. 2:**
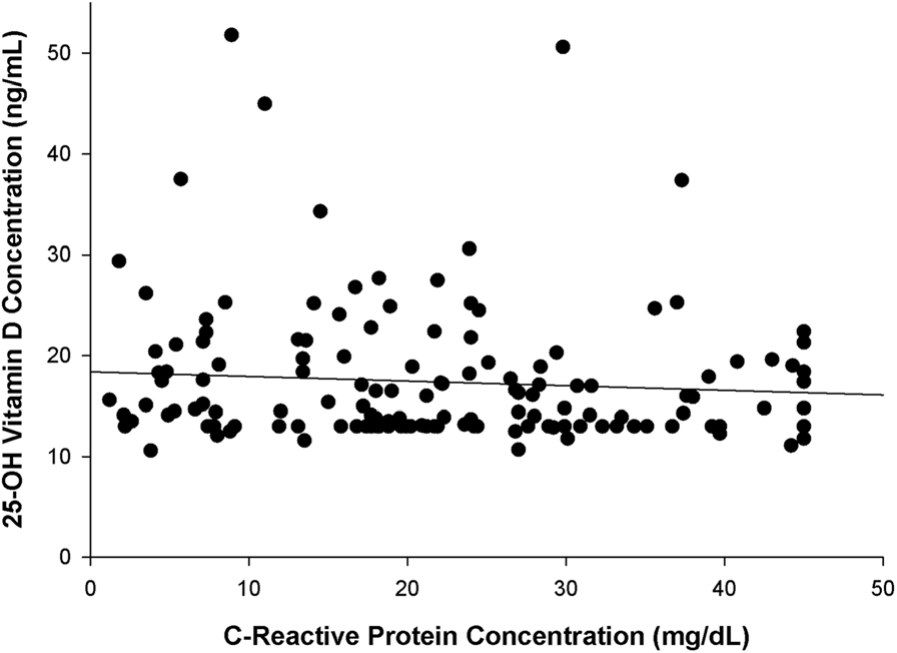
Lack of influence of extent of inflammation as evidenced by C-reactive protein concentration upon serum 25-OH vitamin D concentration (*r* = 0.046, *P* = 0.571). The relationship was described by 25-OH vitamin D concentration = (−0.05 × C-reactive protein concentration) + 18.6

**Fig. 3:**
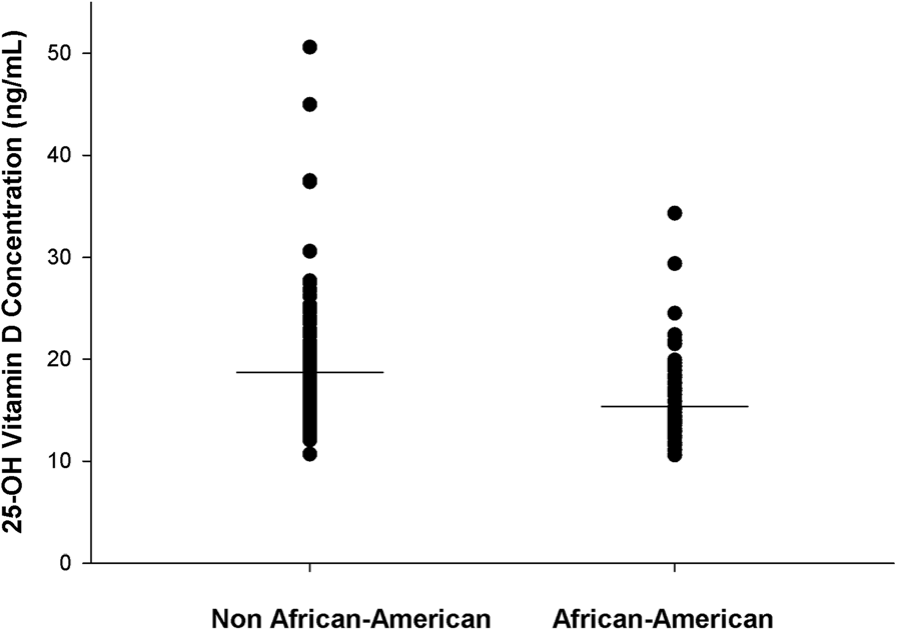
Mean serum 25-OH vitamin D concentration (depicted by the *solid horizontal line*) and range for African-Americans and those of non-African-American race (15.4 ± 4.2 versus 18.7 ± 6.9 ng/mL, respectively, *P* < 0.001)

There were no apparent correlative relationships between serum 25-OH vitamin D concentration and extent of injury as assessed by ISS (*r* = 0.046, *P* = NS, Fig. 1) or between the amount of inflammation present as evaluated by serum C-reactive protein concentration (*r* = 0.096, *P* = NS; Fig. 2). A greater proportion of patients with penetrating injuries (gunshot and knife stab wounds) experienced severe vitamin D deficiency (Table 1; *P* = 0.001). Race influenced serum 25-OH vitamin D concentrations as African-Americans had a significantly lower serum 25-OH vitamin D concentration than non-African-Americans (15.4 ± 4.2 versus 18.7 ± 6.9 ng/mL, *P* = 0.001; Fig. 3) and demonstrated a greater proportion of patients with severe vitamin D deficiency (Table 1; *P* = 0.004). Binomial logistic regression analysis indicated that admitting diagnosis of a gunshot or knife stab wound, African-American race, and increasing body mass index were the most statistically significant covariates associated with vitamin D deficiency (Table 2). Admission diagnosis of a gunshot or knife stab wound was also significantly associated with severe vitamin D deficiency; however, the association with African-American race and body mass index did not achieve statistical significance with severe vitamin D deficiency (Table 2).

**Table 2:** Variables associated with vitamin D deficiency

Vitamin D deficiency and severe deficiency (25-OH vitamin D <20 ng/mL)		
Variable	OR	*P*
GSW/KSW	9.23 (1.13, 75.40)	0.038
African-American race	4.02 (1.40, 11.58)	0.010
Body mass index	1.12 (1.03, 1.23)	0.010
Serum C-reactive protein	1.04 (1.00, 1.08)	0.062
Severe vitamin D deficiency (serum 25-OH vitamin D ≤13 ng/mL)		
Variable	OR	*P*
GSW/KSW	3.52 (1.49, 8.23)	0.004
African-American race	1.88 (0.87, 4.07)	0.109

*GSW* gunshot wound, *KSW* knife stab wound, *OH* hydroxy, *OR* odds ratio

**Fig. 4:**
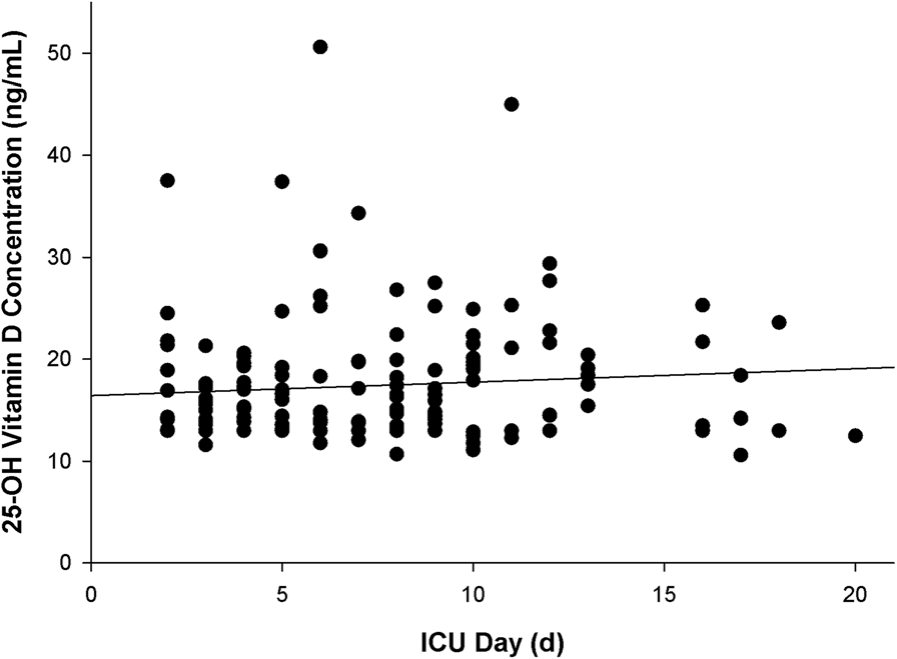
Influence of intensive care unit (ICU) day upon serum 25-OH vitamin D concentration (*r* = 0.081, *P* = 0.315). The insignificant positive relationship was depicted by 25-OH vitamin D concentration = 16.6 + (0.1 × ICU day)

Serum 25-OH vitamin D concentrations were obtained 7.7 ± 5.1 days following admission to the ICU. No significant correlative relationship was observed between serum 25-OH vitamin D concentration and hospital day for its determination (Fig. 4; *r* = 0.081, *P* = 0.315). Serum 25-OH vitamin D concentration in those patients whose measurements were done 2 to 4 days post admission to the ICU (mean 2.9 ± 0.9 days post admission, *n* = 47) were not substantially different from those who were measured at least 5 days following admission to the ICU (mean 9.5 ± 5.9 days post admission, *n* = 111): 16.2 ± 4.5 versus 17.9 ± 6.7 ng/mL for each time group, respectively (*P* = 0.231). Ten patients with vitamin D deficiency or severe deficiency who were receiving maintenance vitamin D therapy from their nutritional formula had a second concentration obtained prior to implementation of enteral vitamin D therapy 10.4 ± 4.6 days after the initial determination. Serum concentrations were minimally changed (14.3 ± 1.9 versus 15.4 ± 2.8 ng/mL, respectively, *P* = 0.158). Similar results were observed for 14 patients with vitamin D insufficiency with a repeated determination 8.3 ± 3.5 days following the initial observation (23.2 ± 3.2 versus 24.2 ± 5.8 ng/mL, *P* = 0.401).

### Clinical outcomes associated with vitamin D deficiency

Clinical outcomes of survival, ICU length of stay, hospital length of stay, and proportion of patients that developed a nosocomial infection (e.g., pneumonia, sepsis, wound infection, intra-abdominal abscess, urinary tract infection) while in the ICU were not statistically significant among groups with varying degrees of vitamin D depletion (Table 3). However, a trend towards a shorter ICU length of stay (mean of 14 versus 21 to 23 days for the other groups, respectively; *P* = 0.053) was observed for those patients who had normal vitamin D stores (Table 4). Unfortunately, clinical outcome comparisons between groups (Table 4) were confounded by provision of supplemental vitamin D therapy in patients with vitamin D deficiency which limited interpretation regarding the clinical impact of vitamin D depletion. However, clinical outcomes among the extent of vitamin D depletion groups were not attributed to any differences in calorie, protein, or intrinsic vitamin D intake from the EN or PN (Table 4).

**Table 3:** Clinical outcomes

Variable	Normal	Insufficient	Deficient	Severely deficient	*P* ≤
25-OH vitamin D concentration range, ng/mL	30–80	20–29.9	13.1–19.9	≤13	
*N*	6	31	75	46	
Survived, *n* (%)	5 (83 %)	27 (87 %)	64 (85 %)	36 (80 %)	0.835
ICU length of stay, days	14 ± 7	23 ± 9	23 ± 18	21 ± 16	0.053
Hospital length of stay, days	22 ± 13	38 ± 20	35 ± 24	38 ± 22	0.132
Infection, *n* (%)	6 (100 %)	28 (90 %)	54 (72 %)	35 (76 %)	0.108
Hospital day of infection, days	6 ± 2	9 ± 7	6 ± 5	8 ± 6	0.215

*ICU* intensive care unit, *N* number of patients, *OH* hydroxy

**Table 4:** Vitamin D intake and nutrition therapy

Variable	Normal	Insufficient	Deficient	Severely deficient	*P* ≤
25-OH vitamin D concentration range, ng/mL	30–80	20–29.9	13.1–19.9	≤13	
*N*	6	31	75	46	
Received supplemental ergocalciferol, *n* (%)	0 (0 %)	5 (16 %)	57 (76 %)	35 (76 %)	0.001
Day ergocalciferol started, days	–	16 ± 4	10 ± 7	11 ± 8	0.286
Ergocalciferol duration, days	–	27 ± 24	15 ± 13	20 ± 17	0.095
Caloric intake, kcals/days	1060 ± 524	1397 ± 473	1386 ± 562	1420 ± 412	0.527
Protein intake, g/days	109 ± 41	114 ± 41	106 ± 41	108 ± 30	0.797
EN/PN, *n*/*n*	6/0	26/5	55/20	33/13	0.526
Vitamin D from EN/PN, IU/day	818 ± 279	953 ± 326	769 ± 316	800 ± 280	0.084

*EN* enteral nutrition, *N* number of patients, *PN* parenteral nutrition, *OH* hydroxy

## Discussion

Vitamin D depletion has been associated with an increased rate of infections, lengthened hospital stay, and worsened mortality for critically ill patients [2–8, 30, 31]. These worsened outcomes were potentially related to the discovery that the synthesis of 1,25 - dihydroxy [(OH)_2_] vitamin D within immune cells derived from circulating 25-OH vitamin D enhances antibacterial activity by triggering production of cathelicidin and other antimicrobial peptides [9, 32, 33]. Thus, the presence of vitamin D deficiency would result in decreased availability of substrate for 1,25 -(OH)_2_ vitamin D synthesis [34] and, ultimately, decreased production of antimicrobial peptides. The objective of this study was to ascertain the prevalence and risk factors for vitamin D deficiency for critically ill patients with severe traumatic injuries who are anticipated to have a prolonged ICU length of stay and who require specialized nutrition therapy.

Our data indicated that the majority (77 %) of critically ill patients with traumatic injuries are either deficient or severely deficient with only 4 % of patients having a normal 25-OH vitamin D serum concentration. Our data also indicated that those with penetrating injuries due to a gunshot or knife stab wound, of African-American descent, or an increased body mass index (i.e., obesity) were at greater risk for exhibiting vitamin D deficiency. Our finding regarding the marked and nearly universal prevalence of vitamin D insufficiency or deficiency was important due to the limited amount of data for critically ill patients with severe traumatic injuries [10, 11, 35].

The etiology for the high incidence of vitamin D deficiency in our population was likely multifactorial. African-Americans have historically been noted to be at risk for vitamin D deficiency due to increased melanin pigment content resulting in a decreased synthesis of pre-vitamin D3 in the skin during exposure to ultraviolet radiation [34]. Recent data also indicate that serum vitamin D-binding protein concentrations are lower in African-Americans than American Caucasians due to genetic polymorphism differences [36]. Vitamin D-binding protein accounts for 85 to 90 % of total circulating 25-OH vitamin D with serum albumin binding accounting for an additional 10 to 15 % [36]. Thus, race-specific vitamin D-binding protein concentrations differences [36, 37], independent of critical illness, may have also contributed at least in part, for the observed significant decrease in serum 25-OH vitamin D concentration for African-Americans in our trauma patient population.

Since humans obtain 40 to 50 % of their vitamin D requirements from ultraviolet radiation exposure to sunlight [38], it would be anticipated that the winter months would result in a higher incidence of vitamin D deficiency due to a decrease in outdoor activity as well as a seasonal change in the angle refraction of light from the sun [11, 20]. Although others suggest a profound 50 % increase in the presence of vitamin D deficiency in critically ill patients when comparing winter month admissions to a late summer month [8], we found only a modest, statistically insignificant, 16 % and 12 % increase in the incidence of vitamin D deficiency and severe deficiency, respectively. These data suggest other factors outside of pre-hospitalization sunlight exposure may be more important in the pathogenesis of vitamin D deficiency for our critically ill population.

Obesity, in health as well as in critical illness, may also play a role in the development of vitamin D insufficiency and deficiency [37, 39–41]. It has been proposed that decreased 25-OH vitamin D concentrations in obese patients occur due to its sequestration within body fat compartments [41] particularly in those with an increased waist to hip ratio [39, 40]. Patients with a normal 25-OH vitamin D had a significantly lower body mass index than the vitamin insufficient and deficient groups (Table 1). The role of obesity in modulating circulating 25-OH vitamin D concentrations during critical illness and its impact upon clinical outcomes warrants further investigation.

There is an abundant amount of emerging literature that critical illness, per se, is a causative factor in the development of vitamin D insufficiency and deficiency [1–8, 10, 11, 29, 30, 35]. Serum 25-OH vitamin D concentrations have been demonstrated to decrease as early as 3 days post ICU admission and remain depressed for at least 10 days [30]. Inflammatory stress states, including sepsis, results in decreases in serum vitamin D-binding protein concentration [1, 8, 42] which ultimately results in a decrease in 25-OH vitamin D concentration. Our population exhibited a high level of stress and inflammation as evidenced by a high ISS and elevated CRP concentrations (Table 1).

The most appropriate time post admission for assessing the presence of vitamin D deficiency for critically ill patients is controversial. Fluid resuscitation may acutely lower 25-OH vitamin D concentration. Serial 25-OH vitamin D concentrations in post cardiopulmonary bypass surgery patients were reduced by 35 % during the procedure but returned to within ~10 % of baseline concentrations within 24 h [43]. Seventy percent of our 25-OH vitamin D observations were conducted at least 5 days after admission to the ICU, and none were measured any earlier than 2 days post admission as we attempted to avoid obtaining serum concentrations during fluid resuscitation.

It also has been recommended that multiple determinations be performed due to assay variability and possible diurnal variation in 25-OH vitamin D concentrations [44]. However, the influence of diurnal variation was eliminated in our study as vitamin D concentrations were obtained at about the same time (0100) for all patients. Twenty-four patients had a second 25-OH vitamin D determination several days later (and prior to supplemental vitamin D therapy [29]) which indicated a clinically irrelevant difference in concentration of ~1 ng/mL. Taken together, these data indicate that the observed high prevalence of vitamin D insufficiency and deficiency for critically ill patients with traumatic injuries, based on serum 25-OH vitamin D concentration, was correctly quantitated.

No statistically significant difference was noted among groups for infection rates; however, it is possible that clinical outcomes in those with vitamin D deficiency may have been confounded by the provision of vitamin D therapy per the current practice of the Nutrition Support Service at our institution (Table 4) [29]. Patients with a normal 25-OH vitamin D concentration and did not require vitamin D supplementation had a decreased ICU length of stay that approached statistical significance (*P* = 0.053) compared to the other groups (Table 3); however, these data should also be interpreted with caution due to the low number of subjects with a normal serum 25-OH vitamin D concentration. Although numerous studies suggest a relationship between vitamin D deficiency and increased mortality and infectious complications for critically ill patients [2–8], it is still considered controversial and unclear whether a low serum 25-OH vitamin D concentration may be a signal of an activated immune system during the acute phase of critical illness rather than a risk factor for impaired immunity or survival [32, 35]. It also has been suggested that it is not entirely clear if a poor vitamin D status is more of an indicator of an unhealthy lifestyle, multiple risk factors for morbidity, and consequently associated with poorer prognostic outcomes [45].

This study has limitations. This study was performed at a single medical center and lacks a comparative normal healthy control group from our region of the country. Pre-morbid vitamin D concentrations for the patients were not available. The assay for vitamin D-binding protein was unavailable at our institution which could have provided further insight into the amount of bioavailable vitamin D with each group. However, lack of vitamin D-binding protein concentrations will not alter the interpretation of these data as 25-OH vitamin D and binding protein concentrations tend to parallel each other [14, 36]. The timing of the initial and subsequent 25-OH vitamin D assays post admission to the ICU was allowed to be within a target range of about 2 weeks post admission (excluding the immediate resuscitation period) and not precisely standardized by day of ICU admission among groups. A greater number of patients with a normal 25-OH vitamin D concentration would have been more meaningful for comparative group evaluations. Finally, comparison of clinical outcomes for the depleted and severely depleted patient groups were likely skewed by the administration of supplemental vitamin D therapy. A separate analysis of groups without provision of vitamin D supplementation is warranted for examining the extent of vitamin D depletion upon clinical outcomes.

## Conclusions

The majority of critically ill patients with traumatic injuries, particularly those with penetrating wounds, and those with obesity or of African-American race are at greater risk for deficiency. Severity of injury, extent of inflammation, or hospital admission during the winter season did not significantly influence the prevalence of vitamin D deficiency. Whether vitamin D supplementation will improve clinical outcomes for this population requires further research.

## Abbreviations

BMI, body mass index; CRP, C-reactive protein; EN, enteral nutrition; GSW, gunshot wound; ICU, intensive care unit; ISS, injury severity score; KSW, knife stab wound; Mar, March; MVA, motor vehicle accident; Nov, November; NS, not significant; NSS, nutrition support service; OR, odds ratio; PN, parenteral nutrition; 25-OH vitamin D, 25-hydroxy vitamin D

## References

[1] Jeng L, Yamshchikov AV, Judd SE, Blumberg HM, Martin GS, Ziegler TR (2009). Alterations in vitamin D status and anti-microbial peptide levels in patients in the intensive care unit with sepsis. J Transl Med.

[2] Braun A, Chang D, Mahadevappa K, Gibbons FK, Liu Y, Giovannucci E (2011). Association of low serum 25-hydroxyvitamin D levels and mortality in the critically ill. Crit Care Med.

[3] Braun AB, Gibbons FK, Litonjua AA, Giovannucci E, Christopher KB (2012). Low serum 25-hydroxyvitamin D at critical care initiation is associated with increased mortality. Crit Care Med.

[4] Braun AB, Litonjua AA, Moromizato T, Gibbons FK, Giovannucci E, Christopher KB (2012). Association of low serum 25-hydroxyvitamin D levels and acute kidney injury in the critically ill. Crit Care Med.

[5] Moromizato T, Litonjua AA, Braun AB, Gibbons FK, Giovannucci E, Christopher KB (2014). Association of low serum 25-hydroxyvitamin D levels and sepsis in the critically ill. Crit Care Med.

[6] Quraishi SA, Bittner EA, Blum L, McCarthy CM, Bhan I, Camargo CA (2014). Prospective study of vitamin D status at initiation of care in critically ill surgical patients and risk of 90-day mortality. Crit Care Med.

[7] Venkatram S, Chilimuri S, Adrish M, Salako A, Patel M, Diaz-Fuentes G (2011). Vitamin D deficiency is associated with mortality in the medical intensive care unit. Crit Care.

[8] Amrein K, Zajic P, Schnedl C, Waltensdorfer A, Fruhwald S, Holl A (2014). Vitamin D status and its association with season, hospital and sepsis mortality in critical illness. Crit Care.

[9] Schwalfenberg GK (2011). A review of the critical role of vitamin D in the functioning of the immune system and the clinical implications of vitamin D deficiency. Mol Nutr Food Res.

[10] Flynn L, Zimmerman LH, McNorton K, Dolman M, Tyburski J, Baylor A (2012). Effects of vitamin D deficiency in critically ill surgical patients. Am J Surg.

[11] Matthews LR, Ahmed Y, Wilson KL, Griggs DD, Danner OK (2012). Worsening severity of vitamin D deficiency is associated with increased length of stay, surgical intensive care unit cost, and mortality rate in surgical intensive care unit patients. Am J Surg.

[12] Klein GL (2011). Burns: where has all the calcium (and vitamin D) gone?. Adv Nutr.

[13] Oleson CV, Patel PH, Wuermser LA (2010). Influence of season, ethnicity, and chronicity on vitamin D deficiency in traumatic spinal cord injury. J Spinal Cord Med.

[14] Lips P, Bouillon R, Jongen MJ, van Ginkel FC, van der Vijgh WJ, Netelenbos JC (1985). The effect of trauma on serum concentrations of vitamin D metabolites in patients with hip fracture. Bone.

[15] Reid D, Toole BJ, Knox S, Talwar D, Harten J, O'Reilly DS (2011). The relation between acute changes in the systemic inflammatory response and plasma 25-hydroxyvitamin D concentrations after elective knee arthroplasty. Am J Clin Nutr.

[16] Magnotti LJ, Croce MA, Fabian TC (2004). Is ventilator-associated pneumonia in trauma patients an epiphenomenon or a cause of death?. Surg Infect (Larchmt).

[17] Theodorou M, Serste T, Van Gossum M, Dewit S (2014). Factors associated with vitamin D deficiency in a population of 2044 HIV-infected patients. Clin Nutr.

[18] Holick MF, Binkley NC, Bischoff-Ferrari HA, Gordon CM, Hanley DA, Heaney RP (2011). Evaluation, treatment, and prevention of vitamin D deficiency: an Endocrine Society clinical practice guideline. J Clin Endocrinol Metab.

[19] Robien K, Oppeneer SJ, Kelly JA, Hamilton-Reeves JM (2013). Drug-vitamin D interactions: a systematic review of the literature. Nutr Clin Pract.

[20] Bandeira F, Griz L, Dreyer P, Eufrazino C, Bandeira C, Freese E (2006). Vitamin D deficiency: a global perspective. Arq Bras Endocrinol Metabol.

[21] Baker SP, O'Neill B, Haddon W, Long WB (1974). The injury severity score: a method for describing patients with multiple injuries and evaluating emergency care. J Trauma.

[22] Dickerson RN, Pitts SL, Maish GO, Schroeppel TJ, Magnotti LJ, Croce MA (2012). A reappraisal of nitrogen requirements for patients with critical illness and trauma. J Trauma Acute Care Surg.

[23] Kudsk KA, Minard G, Croce MA, Brown RO, Lowrey TS, Pritchard FE (1996). A randomized trial of isonitrogenous enteral diets after severe trauma. An immune-enhancing diet reduces septic complications. Ann Surg.

[24] Dickerson RN, Swiggart CE, Morgan LM, Maish GO, Croce MA, Minard G (2008). Safety and efficacy of a graduated intravenous insulin infusion protocol in critically ill trauma patients receiving specialized nutritional support. Nutrition.

[25] Dickerson RN, Wilson VC, Maish GO, Croce MA, Minard G, Brown RO (2013). Transitional NPH insulin therapy for critically ill patients receiving continuous enteral nutrition and intravenous regular human insulin. JPEN J Parenter Enteral Nutr.

[26] Dickerson RN, Lynch AM, Maish GO, Croce MA, Minard G, Brown RO (2014). Improved safety with intravenous insulin therapy for critically ill patients with renal failure. Nutrition.

[27] Farrell CJ, Martin S, McWhinney B, Straub I, Williams P, Herrmann M (2012). State-of-the-art vitamin D assays: a comparison of automated immunoassays with liquid chromatography-tandem mass spectrometry methods. Clin Chem.

[28] 25-OH vitamin D Architect System package insert. Abbott Laboratories. Abbott Park, IL; 2011.

[29] Dickerson RN, Berry SC, Ziebarth JD, Swanson JM, Maish GO, Minard G (2015). Dose-response effect of ergocalciferol therapy on serum 25-hydroxyvitamin D concentration during critical illness. Nutrition.

[30] Higgins DM, Wischmeyer PE, Queensland KM, Sillau SH, Sufit AJ, Heyland DK (2012). Relationship of vitamin D deficiency to clinical outcomes in critically ill patients. JPEN J Parenter Enteral Nutr.

[31] Blay B, Thomas S, Coffey R, Jones LM, Murphy C (2016). Evaluation of vitamin D status in burn injured patients. J Burn Care Res.

[32] Kempker JA, Tangpricha V, Ziegler TR, Martin GS (2012). Vitamin D in sepsis: from basic science to clinical impact. Crit Care.

[33] Han JE, Ziegler TR (2014). Vitamin D supplementation in sepsis and critical illness: where are we now?. Am J Respir Crit Care Med.

[34] Holick MF (2007). Vitamin D deficiency. N Engl J Med.

[35] Cecchi A, Bonizzoli M, Douar S, Mangini M, Paladini S, Gazzini B (2011). Vitamin D deficiency in septic patients at ICU admission is not a mortality predictor. Minerva Anestesiol.

[36] Powe CE, Evans MK, Wenger J, Zonderman AB, Berg AH, Nalls M (2013). Vitamin D-binding protein and vitamin D status of black Americans and white Americans. N Engl J Med.

[37] Shafinaz IS, Moy FM (2016). Vitamin D level and its association with adiposity among multi-ethnic adults in Kuala Lumpur, Malaysia: a cross sectional study. BMC Public Health.

[38] Rosen CJ (2011). Clinical practice. Vitamin D insufficiency. N Engl J Med.

[39] Andreozzi P, Verrusio W, Viscogliosi G, Summa ML, Gueli N, Cacciafesta M (2016). Relationship between vitamin D and body fat distribution evaluated by DXA in postmenopausal women. Nutrition.

[40] Gangloff A, Bergeron J, Pelletier-Beaumont E, Nazare JA, Smith J, Borel AL (2015). Effect of adipose tissue volume loss on circulating 25-hydroxyvitamin D levels: results from a 1-year lifestyle intervention in viscerally obese men. Int J Obes (Lond).

[41] Wortsman J, Matsuoka LY, Chen TC, Lu Z, Holick MF (2000). Decreased bioavailability of vitamin D in obesity. Am J Clin Nutr.

[42] Duncan A, Talwar D, McMillan DC, Stefanowicz F, O'Reilly DS (2012). Quantitative data on the magnitude of the systemic inflammatory response and its effect on micronutrient status based on plasma measurements. Am J Clin Nutr.

[43] Krishnan A, Ochola J, Mundy J, Jones M, Kruger P, Duncan E (2010). Acute fluid shifts influence the assessment of serum vitamin D status in critically ill patients. Crit Care.

[44] Venkatesh B, Davidson B, Robinson K, Pascoe R, Appleton C, Jones M (2012). Do random estimations of vitamin D3 and parathyroid hormone reflect the 24-h profile in the critically ill?. Intensive Care Med.

[45] Amrein K, Christopher KB, McNally JD (2015). Understanding vitamin D deficiency in intensive care patients. Intensive Care Med.

